# A Rare Case of Congenital Diabetes Insipidus

**DOI:** 10.3389/fmed.2015.00043

**Published:** 2015-07-07

**Authors:** Tanvi Rege, Srujana Polsani, Belinda Jim

**Affiliations:** ^1^Nephrology Division, Department of Medicine, Jacobi Medical Center, Albert Einstein College of Medicine, Bronx, NY, USA; ^2^University Medical Center of Princeton at Plainsboro, Rutgers Robert Wood Johnson Medical School, Plainsboro, NJ, USA

**Keywords:** congenital, genetic, diabetes insipidus, vasopressin, antidiuretic hormone, vasopressin receptor, aquaporin

## Abstract

Congenital nephrogenic diabetes insipidus (NDI) is a conformation disease resulting from protein misfolding. Ninety percent of mutations result from the inactivating mutations of the arginine vasopressin receptor 2 (AVPR2) gene transmitted in an X-linked fashion, blocking the response to vasopressin, resulting in the inability to concentrate urine. Clinical features include polyuria, polydispsia, dehydration, and hypernatremia. They are generally more severely in affected males but present variably in females due to skewed inactivation of the X chromosome. We describe a case of a 40-year-old woman with a history of Type 2 diabetes mellitus, hyperlipidemia, and obesity, who presents with debilitating polyuria since the age of 5 with no clear diagnosis. Interestingly, her son was diagnosed with NDI. Genetic testing revealed that she was heterozygous for the Val88Met mutation in the AVPR2 gene while her son was hemizygous for the same. The patient has since been successfully treated with diuretics and a low solute diet. We highlight that although X-linked NDI patients are mostly males, it should be considered in symptomatic females to prevent delays in the diagnosis. Conformational diseases such as NDI are presently the subject of research using pharmacological chaperones to restore proper receptor membrane localization and function.

## Case Report

We describe a 40-year-old Hispanic female with a history significant for diabetes mellitus Type 2, hyperlipidemia, and obesity, who was referred to the nephrology clinic for evaluation of polyuria. Her family history was significant for diabetes insipidus diagnosed in her son at 8 years of age. This patient has been evaluated for polyuria and polydipsia since the age of 5 years with no definite diagnosis. She reports drinking 4–5 gallons of water every day due to extreme thirst. She also admits to debilitating nocturia, urinating about 10 times every night.

Her laboratory tests revealed a serum sodium in the range of 138–145 mEq/L, serum osmolality of 286 mOsm/kg, with a concomitant urine osmolality of 171 mOsm/kg. The patient’s hemoglobin_A1C_ was elevated at 8.9, though her urine did not reveal glucosuria. Her urine osmolality ranged from 72 to 171 mOsm/kg measured on multiple occasions. Her urinalysis was also significant for proteinuria with a urine protein-to-creatinine ratio ranging from 400 to 800 mg/g. Urine electrolytes revealed a urine sodium of <20 mmol/L, urine chloride of 20 mmol/L, and urine potassium of 50 mmol/L. The patient’s CT scan of the abdomen was consistent with a recently passed stone.

Given the patient’s strong family history and findings, the patient and her son underwent genetic testing. The patient was found to be heterozygous for Val88Met mutation in the AVPR2 gene while her son was found to be hemizygous for the same.

The patient has since been successfully treated with hydrochlorothiazide (HCTZ) at 12.5 mg once daily and a low solute diet with significant improvement in her symptoms.

This patient has given her consent to report her case in a publication.

## Discussion

Nephrogenic diabetes insipidus (NDI) is a rare genetic disease caused most commonly by mutations in two different genes. Though the exact prevalence is unknown, Arthus et al. found that the prevalence of NDI in Quebec, Canada to be 8.8 per 1,000,000 males. Interestingly, the Nova Scotia and New Brunswick population exhibited an incidence that was six times higher (1).

In 90% of patients, the affected gene is arginine vasopressin receptor 2 (AVPR 2), whereas the remainder of patients have been shown to have had a defect in the aquaporin 2 (AQP2) gene (2). Both of these receptors play a crucial role in the concentration of urine by facilitating water reabsorption through the action of anti-diuretic hormone (ADH), also known as vasopressin.

The AVPR2 gene is located in the chromosome region Xq28; it has three exons and two introns and is inherited in an X-linked mode of transmission (3). This gene encodes for vasopressin 2 (V2) receptors located in the basolateral membrane of the principal cell. The V2 receptor is a three-dimensional protein consisting of 371 amino acid residues that form 7 transmembrane, 4 extracellular, and 4 cytoplasmic domains (4). These receptors mediate the action of vasopressin in the collecting ducts. Binding to vasopressin leads to the formation of cAMP, which activates protein kinase and initiates a phosphorylation cascade; this results in the exocytic insertion AQP2receptors in the luminal membrane, thereby increasing water permeability and reabsorption (5).

More than 200 mutations of the AVPR2 gene have been reported in the Human Gene Mutation Database (6). Missense mutations were responsible for 55.83%, of which the amino acids arginine and tyrosine were most commonly mutated (7). The majority of the affected individuals are diagnosed in the first year of life, whereas those with partial and heterozygote for X-linked NDI may present with symptoms later in life (8).

A study from Montreal of 90 families with AVPR2 mutations demonstrated that most have a full phenotype, while only three families have a mild version (9). In males with the AVPR2 mutation, the phenotype is characterized by severe dehydration, hypernatremia, hyperthermia, mental and physical retardation, and kidney failure. Women with this genetic defect are heterozygous and exhibit variable degrees of clinical symptoms. This varied phenotypic expression may be related to the skewed inactivation of X chromosome (10).

Our patient harbors the V88M mutation. V88M mutation produces V2 receptors which are trapped intracellularly. Pan et al. initially described V88M as a sequence variant (11) but was later regarded V88M as a disease causing mutation (12). Arthus et al. studied two severely affected females with the V88M mutation who demonstrated extreme skewing in favor of the paternal X chromosome (1). The study also estimated that about 1% of females who is heterozygous for the AVPR2 mutation may exhibit a phenotype as severe as that of NDI males. He concluded that although X-linked NDI patients are mostly males, it should also be included in the differential diagnosis of symptomatic females to prevent delays in the diagnosis.

The AQP2 gene, on the other hand, is located on chromosome region 12q13 and has 4 exons and 3 introns. The gene codes for a polypeptide of 271 amino acids and has six membrane spanning domains (4). The AQP2 water channel usually lies dormant in the intracellular vesical membranes. Upon binding of vasopressin to the V2 receptor, it fuses with the luminal membrane of the principal cells. About 35 mutations in the AQP2 gene have been reported that follow an autosomal recessive or autosomal dominant mode of inheritance. Mutations in the recessive form of NDI are located throughout the gene, resulting in misfolded proteins that are retained in the endoplasmic reticulum. On the contrary, mutations in the dominant form of this disorder are localized to the carboxyl terminus of gene and the resultant AQP2 mutants are properly folded, but missorted (13). The dominant form of inheritance is known to have mild clinical manifestations as compared to the recessive form (6).

Renal ultrasound is recommended to evaluate for hydronephrosis, dilatation of the urinary tract, and megacystitis. Urinary tract dilatation is a well-known but rare complication attributed mainly to the large volume of urine produced. Polyuria usually causes no more than mild to moderate hydronephrosis; however, rare cases of severe urinary tract dilatation have been reported (14). If a disease causing mutation has been identified in a family, then appropriate genetic testing and counseling should be offered to symptomatic individuals and high risk pregnancies.

General management of NDI involves a low salt, low protein diet with administration of diuretics and non-steroidal anti-inflammatory drugs (NSAIDS). Paradoxically, thiazide diuretics can cause a state of mild volume contraction so that more sodium and water can be reabsorbed in the proximal tubules and less water is delivered to the vasopressin-sensitive site of the principal cell (15). Prostaglandins may alleviate symptoms by reducing the glomerular filtration rate, increasing proximal tubule or distal tubule sodium reabsorption, and exerting an inhibitory effect on the action of vasopressin (16, 17). A study that compared the treatment with HCTZ alone, HCTZ/triamterene, HCTZ/amiloride, and HCTZ/acemetacin (NSAID) demonstrated the superiority of the HCTZ/amiloride combination in that it prevented hypokalemia and metabolic alkalosis (18). In infants, special attention must be paid to minimize polyuria, hypernatremia, and volume depletion. In adults, treatment depends on the clinical symptoms of polyuria since electrolyte abnormalities and volume depletion are generally protected by the intact thirst mechanism. In patients complicated by both diabetes mellitus and insipidus, as in our patient, it may be difficult to discriminate between which entity is truly contributing intractable polyuria. Thus, the first step is to achieve strict glycemic control to avoid the exacerbation of unmitigated glucosuria before employing strategies to treat NDI.

Since the cloning of the V2R gene in 1992 (3), developing the use of chaperones, a rescue mechanism to correct mutations, is possible. Chaperones are pharmacological ligands, which act by binding to and stabilizing specific conformations of their receptors. To this end, Morello and colleagues have successfully used vasopressin analogs capable of acting as chaperones to rescue misfolded mutant V2 receptors to their proper folding and function (19). A recent human pilot trial also showed that a low-affinity agent for V2R significantly decreased the 24 h urine volume and water intake (20). Unfortunately, due to the potential interference with the cytochrome P450 metabolic pathway and hepatic toxicity, this trial was terminated at the phase II level. Despite this, the concept appears to be valid and research using this “chaperone” method continues.

To conclude, congenital NDI is a rare genetic disorder in which the majority of the affected are males who are diagnosed during infancy. However, carrier females have variable phenotype causing them to remain undiagnosed early in life. Their quality of life could be affected from distressing symptoms like extreme polyuria and polydipsia, putting them at a risk for dehydration. Strong suspicion should be maintained in the setting of classical symptoms and family history. Genetic testing should be encouraged to confirm the diagnosis. At present, management of these patients includes correcting of confounding diagnoses, adequate water intake, use of diuretics and NSAIDs, as well as a low solute diet.

## Conflict of Interest Statement

The authors declare that the research was conducted in the absence of any commercial or financial relationships that could be construed as a potential conflict of interest.

## References

[1] Arthus MF Lonergan M Crumley MJ Naumova AK Morin D De Marco LA Report of 33 novel AVPR2 mutations and analysis of 117 families with X-linked nephrogenic diabetes insipidus. J Am Soc Nephrol (2000) 11(6):1044–54. 10820168 10.1681/ASN.V1161044

[2] Sasaki S Chiga M Kikuchi E Rai T Uchida S. Hereditary nephrogenic diabetes insipidus in Japanese patients: analysis of 78 families and report of 22 new mutations in AVPR2 and AQP2. Clin Exp Nephrol (2013) 17(3):338–44. 10.1007/s10157-012-0726-z 23150186

[3] Birnbaumer M Seibold A Gilbert S Ishido M Barberis C Antaramian A Molecular cloning of the receptor for human antidiuretic hormone. Nature (1992) 357(6376):333–5. 10.1038/357333a0 1534149

[4] Fujiwara TM Bichet DG. Molecular biology of hereditary diabetes insipidus. J Am Soc Nephrol (2005) 16(10):2836–46. 10.1681/ASN.2005040371 16093448

[5] Brown D. The ins and outs of aquaporin-2 trafficking. Am J Physiol Renal Physiol (2003) 284(5):F893–901. 10.1152/ajprenal.00387.2002 12676734

[6] Shida Y Matsuoka H Chiga M Uchida S Sasaki S Sugihara S. Characterization of AQP-2 gene mutation (R254Q) in a family with dominant nephrogenic DI. Pediatr Int (2013) 55(1):105–7. 10.1111/j.1442-200X.2012.03614.x 23409988

[7] Spanakis E Milord E Gragnoli C. AVPR2 variants and mutations in nephrogenic diabetes insipidus: review and missense mutation significance. J Cell Physiol (2008) 217(3):605–17. 10.1002/jcp.21552 18726898

[8] van Lieburg AF Knoers NV Monnens LA. Clinical presentation and follow-up of 30 patients with congenital nephrogenic diabetes insipidus. J Am Soc Nephrol (1999) 10(9):1958–64. 10477148 10.1681/ASN.V1091958

[9] Bichet DG Oksche A Rosenthal W Congenital nephrogenic diabetes insipidus. J Am Soc Nephrol (1997) 8(12):1951–8. 9402099 10.1681/ASN.V8121951

[10] Amos-Landgraf JM Cottle A Plenge RM Friez M Schwartz CE Longshore J X chromosome-inactivation patterns of 1,005 phenotypically unaffected females. Am J Hum Genet (2006) 79(3):493–9. 10.1086/507565 16909387 PMC1559535

[11] Pan Y Metzenberg A Das S Jing B Gitschier J. Mutations in the V2 vasopressin receptor gene are associated with X-linked nephrogenic diabetes insipidus. Nat Genet (1992) 2(2):103–6. 10.1038/ng1092-103 1303257

[12] Bichet DG Birnbaumer M Lonergan M Arthus MF Rosenthal W Goodyer P Nature and recurrence of AVPR2 mutations in X-linked nephrogenic diabetes insipidus. Am J Hum Genet (1994) 55(2):278–86. 8037205 PMC1918376

[13] Kuwahara M Iwai K Ooeda T Igarashi T Ogawa E Katsushima Y Three families with autosomal dominant nephrogenic diabetes insipidus caused by aquaporin-2 mutations in the C-terminus. Am J Hum Genet (2001) 69(4):738–48. 10.1086/323643 11536078 PMC1226060

[14] Shalev H Romanovsky I Knoers NV Lupa S Landau D. Bladder function impairment in aquaporin-2 defective nephrogenic diabetes insipidus. Nephrol Dial Transplant (2004) 19(3):608–13. 10.1093/ndt/gfg574 14767016

[15] Magaldi AJ New insights into the paradoxical effect of thiazides in diabetes insipidus therapy. Nephrol Dial Transplant (2000) 15(12):1903–5. 10.1093/ndt/15.12.1903 11096127

[16] Knoers N Monnens LA Amiloride-hydrochlorothiazide versus indomethacin-­hydrochlorothiazide in the treatment of nephrogenic diabetes insipidus. J Pediatr (1990) 117(3):499–502. 10.1016/S0022-3476(05)81106-0 2391611

[17] Chevalier RL Rogol AD Tolmetin sodium in the management of nephrogenic diabetes insipidus. J Pediatr (1982) 101(5):787–9. 10.1016/S0022-3476(82)80322-3 6957583

[18] Konoshita T Kuroda M Kawane T Koni I Miyamori I Tofuku Y Treatment of congenital nephrogenic diabetes insipidus with hydrochlorothiazide and amiloride in an adult patient. Horm Res (2004) 61(2):63–7. 10.1159/000075241 14646392

[19] Morello JP Salahpour A Laperriere A Bernier V Arthus MF Lonergan M Pharmacological chaperones rescue cell-surface expression and function of misfolded V2 vasopressin receptor mutants. J Clin Invest (2000) 105(7):887–95. 10.1172/JCI8688 10749568 PMC377482

[20] Bernier V Morello JP Zarruk A Debrand N Salahpour A Lonergan M Pharmacologic chaperones as a potential treatment for X-linked nephrogenic diabetes insipidus. J Am Soc Nephrol (2006) 17(1):232–43. 10.1681/ASN.2005080854 16319185

